# A New Role of OmpR in Acid and Osmotic Stress in *Salmonella* and *E. coli*

**DOI:** 10.3389/fmicb.2018.02656

**Published:** 2018-11-22

**Authors:** Smarajit Chakraborty, Linda J. Kenney

**Affiliations:** ^1^Mechanobiology Institute, National University of Singapore, Singapore, Singapore; ^2^Department of Biochemistry, National University of Singapore, Singapore, Singapore; ^3^Departments of Microbiology and Immunology, University of Illinois at Chicago, Chicago, IL, United States; ^4^Bioengineering, University of Illinois at Chicago, Chicago, IL, United States; ^5^Jesse Brown Veterans Administration Medical Center, Chicago, IL, United States

**Keywords:** single cells, fluorescence microscopy, two-component regulatory systems, EnvZ, OmpR, GltA, acid stress, osmotic stress

## Abstract

Bacteria survive and respond to diverse environmental conditions and during infection inside the host by systematic regulation of stress response genes. *E. coli* and *S*. Typhimurium can undergo large changes in intracellular osmolality (up to 1.8 Osmol/kg) and can tolerate cytoplasmic acidification to at least pH_i_ 5.6. Recent analyses of single cells challenged a long held view that bacteria respond to extracellular acid stress by rapid acidification followed by a rapid recovery. It is now appreciated that both *S*. Typhimurium and *E. coli* maintain an acidic cytoplasm through the actions of the outer membrane protein regulator OmpR via its regulation of distinct signaling pathways. However, a comprehensive comparison of OmpR regulons between *S*. Typhimurium and *E. coli* is lacking. In this study, we examined the expression profiles of wild-type and *ompR* null strains of the intracellular pathogen *S*. Typhimurium and a commensal *E. coli* in response to acid and osmotic stress. Herein, we classify distinct OmpR regulons and also identify shared OmpR regulatory pathways between *S*. Typhimurium and *E. coli* in response to acid and osmotic stress. Our study establishes OmpR as a key regulator of bacterial virulence, growth and metabolism, in addition to its role in regulating outer membrane proteins.

## Introduction

Eukaryotic cells maintain strict pH homeostasis between pH 7.0–7.4 by ion transport mechanisms and a high buffering capacity of the cytosol (see (Madshus, 1988; Casey et al., 2010) for reviews). Recent advances in fluorophores, imaging technology and the ability to examine single cell behavior has led to a new view of the bacterial response to acid and osmotic stress (Chakraborty et al., 2015, 2017). Unlike eukaryotes, it is now appreciated that bacteria can survive and respond to diverse environmental conditions both inside and outside of the host by systematic regulation of stress response genes (Boor, 2006; Krulwich et al., 2011). The long held view was that Gram negative bacteria such as *E. coli* and *S*. Typhimurium were neutralophiles, i.e., they maintain their intracellular pH between 7.2 and 7.8 (see Slonczewski et al., 2009; De Biase and Lund, 2015 for reviews). For strong acids and pH values down to ∼5.0, it was reported that the periplasm rapidly equilibrated with the external medium, but the cytoplasm showed only transient acidification before returning to its normal pH of ∼7.4 (Wilks and Slonczewski, 2007; Slonczewski et al., 2009). In contrast, recent studies in single cells using fluorescence microscopy and the pH-sensitive fluorophore BCECF-AM reported that *E. coli* and *S*. Typhimurium were acidified in response to both acid and osmotic stress and acidification was maintained for > 90 min (Chakraborty et al., 2017). *In vivo* measurements of *S*. Typhimurium inside macrophage vacuoles using a FRET DNA biosensor termed the I-switch, also reported prolonged acidification (Chakraborty et al., 2015). The mechanism is now established: the global regulator OmpR (best known for its regulation of outer membrane proteins) plays a central role in the bacterial response to acid and osmotic stress (Stincone et al., 2011; Quinn et al., 2014; Chakraborty et al., 2015, 2017), resulting in a substantial reprograming of the bacterial transcriptome. The observation that the cytoplasm was acidified as a consequence of both acid and osmotic stress (Chakraborty et al., 2017), also explains why previous studies reported that acid-induced genes were also induced in response to osmotic stress (De Biase et al., 1999; Kitko et al., 2010).

Environmental stress pathways in bacteria, including acid and osmotic stress, are regulated by two-component regulatory systems (TCRS). These TCRSs employ a sensor histidine kinase, which is most often embedded in the cytoplasmic membrane. The second component is a response regulator, which usually functions as a transcription factor that binds DNA and regulates transcription. The two components communicate via a series of phosphorylation reactions involving autophosphorylation of the kinase on a conserved histidine residue, followed by phosphoryl transfer to an aspartate on the response regulator. The EnvZ/OmpR TCRS is best known for its role in regulating expression of outer membrane porins OmpF and OmpC in response to osmotic stress (Walthers et al., 2005; Anand and Kenney, unpublished).

In most cases, the signaling process by histidine kinases is not well understood. However, in the case of the EnvZ kinase, previous studies using hydrogen-deuterium exchange mass spectrometry established that the sensor was a seventeen amino acid peptide that flanked the phosphorylated histidine (Wang et al., 2012). It was surprising that the sensor was located in the cytoplasm and the cytoplasmic domain alone (EnvZc) was capable of sensing without being in the membrane (Wang et al., 2012), although the presence of the transmembrane domains increased the dynamic range of the response (Ghosh et al., 2017). These studies provided a new view of cytoplasmic signaling by histidine kinases (Wang et al., 2012; Foo et al., 2015a), which led to the proposal that intracellular acid stress was a driver of metabolic reprogramming in response to both acid and osmotic stress (Chakraborty et al., 2015, 2017). More recently, others have shown that additional histidine kinases are capable of intracellular signaling, although most studies are still lacking in mechanistic detail (Eguchi and Utsumi, 2014; Choi and Groisman, 2016; Sen et al., 2017).

During acid stress in *S*. Typhimurium and *E. coli*, OmpR contributes to cytoplasmic acidification by repressing the *cadC/BA* operon (Chakraborty et al., 2015, 2017). CadC is in the OmpR subfamily of response regulators and normally it activates transcription of *cadBA*. CadA is a lysine decarboxylase, which consumes a proton during decarboxylation. The product, cadaverine, is then transported out of the cell by the CadB antiporter. Repression of *cadC/BA* by OmpR thus prevents neutralization. Because the pH optima of the CAD system is 6.1–6.5 (Cheeseman and Fuller, 1968), it is the most important acid stress system when *S*. Typhimurium is in the vacuole during infection inside the host (Chakraborty et al., 2015).

OmpR also promotes acidification in response to osmotic stress, but different pathways are involved. In *S*. Typhimurium, OmpR represses the alternative stationary phase sigma factor, *rpoS*, relieving RpoS repression of *yghA*. YghA is a putative oxidoreductase that is predicted to produce protons (Chakraborty et al., 2017). In *E. coli*, the intracellular pH was less acidic and OmpR regulated different pathways. In *E. coli*, OmpR represses *speF*, the ornithine decarboxylation system, which has a higher pH optimum of 7 (Vivijs et al., 2016) compared to the glutamate and arginine decarboxylation systems (pH optima 4 and 5, respectively) (Bearson et al., 2009). Normally, ornithine decarboxylase decarboxylates arginine, consumes protons and produces putrescine, which allows for recovery from acidification. Repression of *speF* by OmpR prevents recovery at high osmolality (Chakraborty et al., 2017).

In *E. coli* MG1655, whole-genome expression profiling identified acid-responsive genes including: chaperones, regulators and genes involved in metabolism (e.g., glutamine decarboxylase) and some genes associated with the cell envelope. (Tucker et al., 2002). In another study, the *E. coli* response to mild and strong acidic conditions was compared, revealing a complex transcriptional program that was dependent on OmpR and the switch between aerobic and anaerobic growth (Stincone et al., 2011). OmpR was connected to genes involved in pyruvate metabolism and glycolysis, signal transduction and transport and some components of the glutamate decarboxylation (GAD) system. Direct OmpR targets were not identified, most likely because OmpR regulation of acid stress pathways occurs most commonly via repression of transcription, which has less precise DNA recognition requirements (Chakraborty et al., 2015, 2017). Identification of OmpR binding sites by sequence gazing is difficult, because OmpR has a high non-specific DNA binding component, and makes more phosphate backbone contacts and fewer DNA base contacts than other response regulators (Rhee et al., 2008). Since OmpR showed distinct and differential regulation in response to exogenous stresses, in the present work, we set out to elucidate a comprehensive network of OmpR regulons. We compared the gene expression profiles of wild-type and *ompR* null strains between the pathogen *S.* Typhimurium and a non-pathogenic *E. coli* in response to both acid and osmotic stress. Our analysis of OmpR-regulated genes indicates that it drives a major reprogramming in bacteria in response to acid and osmotic stress.

## Materials and Methods

### Bacterial Strains and Growth Conditions

*Salmonella enterica* serovar Typhimurium 14028s and *E. coli* MG1655 were used throughout this study. To determine the acid and osmotic stress response, bacterial strains were grown in a modified N-minimal medium (MgM), buffered with either 100 mM Tris (pH 7.2 ± 15% (w/v) sucrose) or 100 mM MES (pH 5.6), containing 7.5 mM (NH_4_)_2_SO_4_, 5 mM KCl, 0.5 mM K_2_SO_4_, 1 mM KH_2_PO_4_, 10 mM MgCl_2_, 2 mM glucose, and 0.1% Casamino acids. To obtain the growth profiles, cultures of *S*. Typhimurium and *E. coli* in Luria Broth (LB) were grown overnight and sub-cultured (1:100) in 5 ml of MgM pH 7.2 for 24 h. The cultures were then sub-cultured (1:50) in either MgM pH 5.6 or pH 7.2 and incubated for an additional 10 h. The optical density at 600 nm was measured hourly and plotted as a function of time (*n* = 3). The doubling time (Td) was determined by the exponential curve fitting function to generate an equation in the form y = Ae^Bx^, where A, B are numbers and x is the time between doubling of y(A_600nm_). The equation simplifies to Td = *ln2*/B = 0.693/B.

### RNA Isolation and qRT-PCR

Wild-type strains of *E. coli* and *S*. Typhimurium were grown in MgM pH 7.2 for 24 h, then sub-cultured (1:50) in MgM pH 5.6, 7.2, and 7.2 with 15% (w/v) sucrose for 5–6 h until the optical density at 600 nm was ∼0.6. Total RNA was isolated, followed by cDNA synthesis and quantification. The mRNA expression level of the target genes was normalized relative to 16S rRNA.

### Construction of a *gltA* Mutant and Over-Expression Strain

The chromosomal copy of *gltA* from wild-type or an *ompR* null strain of *S*. Typhimurium and *E. coli* was replaced by *tetRA* using λ-Red recombination techniques (Karlinsey, 2007). The *gltA* over-expressed strains were generated by cloning *gltA* into plasmid pMPMA5omega placed under control of the arabinose-inducible pBAD promoter by using primer pairs: *gltA* EcoR1#1F and *gltA* HindIII#1R. The primers used are listed in Supplementary Table S5.

### Fluorescence Measurements of BCECF in *S*. Typhimurium and *E. coli*

Cultures of *S*. Typhimurium and *E. coli* were pre-incubated with 20 μM BCECF-AM for 60 min before the shift to acidic pHe 5.6, neutral pHe 7.2, or high osmolality pHe 7.2 plus 15% (w/v) sucrose as described in Chakraborty et al. (2015, 2017). Cells were placed on microscope slides (Marienfeld) coated with 1% (w/v) agarose and images were analyzed by ImageJ version 1.42.

### Microarray

100 ng of total RNA was labeled with Low Input Quick AMP WT Labeling Kit (Agilent; One-color) following the manufacturer’s instructions. Briefly, 100 ng of total RNA was converted into double-stranded cDNA by priming with an oligo-dT primer containing the recognition site for T7 RNA polymerase. *In vitro* transcription with T7 RNA polymerase was used to produce cyanine 3-CTP labeled cRNA. 600 ng of labeled cRNA was hybridized onto an Agilent SurePrint HD GE 8X15 Microarray (*E. coli*) or Agilent SurePrint G3 custom GE 8X60 Microarray (*S*. Typhimurium) for 17 h at 65°C, 10 rpm in an Agilent hybridization oven. After hybridization, the microarray slide was washed in gene expression buffer 1 (Agilent wash buffer kit) for 1 min at RT and another minute in gene expression buffer 2 (Agilent wash buffer kit) at 37°C before scanning on an Agilent High Resolution Microarray Model C Scanner. The results of gene expression profiles are accessible on the Gene Expression Omnibus (GEO) platform with accession number Microarray data GEO submission GSE106629.

### Data Analysis

DAVID Functional Annotation was used to identify gene clusters, which measures relationships among the annotation terms based on the degrees of their co-association genes to different groups. The “Category” column in Supplementary Table S2B represents the original database or resource where the term originates. DAVID consists of a total of 14 annotation categories, which are all collected in the DAVID knowledgebase such as Gene ontology, Biological process, Molecular function, Cellular component, KEGG pathway, Biocarta pathway, Up keywords, BBID pathway, SMART domain, NIH genetic association, UNIPROT sequence feature, KOG ontology, NCBI OMIM and the Interpro domain. The Kyoto Encyclopedia of Genes and Genome (KEGG) is one such database. The next column in Supplementary Table S2B, “Term,” represents the pathway maps to facilitate biological interpretation in a network context. “Count” signifies the number of genes involved in the process. The *P*-value indicates the threshold of EASE Score, a modified Fisher Exact *P*-Value, for gene-enrichment analysis. Fisher Exact *P*-Value = 0 represents perfect enrichment. *P*-Value ≤ 0.05 is considered to be strongly enriched. “Benjamini” is one of the multiple testing correction techniques used to control family-wide false discovery rate. Functional Annotation Clustering integrates the same techniques of Kappa statistics to measure the degree of the common genes between two annotations. Based on Kappa statistics, more common genes are likely to be grouped together in one cluster. Thus, the presence of genes in more than one cluster represents multiple functions or association between two networks. The non-cluster category in Supplementary Table S2B represents multiple genes having varying biological functions, which cannot be put under one single cluster. The non-cluster generally represents the total pool of genes. Significance analysis was performed using Student’s *t*-test with Benjamini-Horchberg False Discovery Rate (FDR) correction and fold change analysis. The comparison involving 3 conditions was analyzed with the Analysis of Variance (ANOVA) with FDR correction, followed by a *post hoc* testing (Turkey HSD test) and fold change analysis.

### Atomic Force Microscopy

A 703 base pair (bp) region from the *gltA* promoter was gel-purified using the QIAquick Gel Extraction Kit (Qiagen) by using primer pairs *gltA* F and *gltA* R for *S*. Typhimurium (-689 bp to + 14 bp) and for *E. coli* (-694 bp to + 9 bp), respectively. A glutaraldehyde-modified mica surface was prepared as described in Chakraborty et al. (2017). Ten nanograms of the *gltA* regulatory region was incubated with 30 nM OmpR for 15 min at RT. This mixture was then deposited on the mica for 15 min. Images were acquired on a Bruker Dimension FastScan AFM system using the tapping mode with a silicon nitride cantilever (FastScan C, Bruker). Raw AFM images were processed using Gwyddion software^1^.

## Results

### Acid Stress Does Not Affect Cell Growth

**FIGURE 1:**
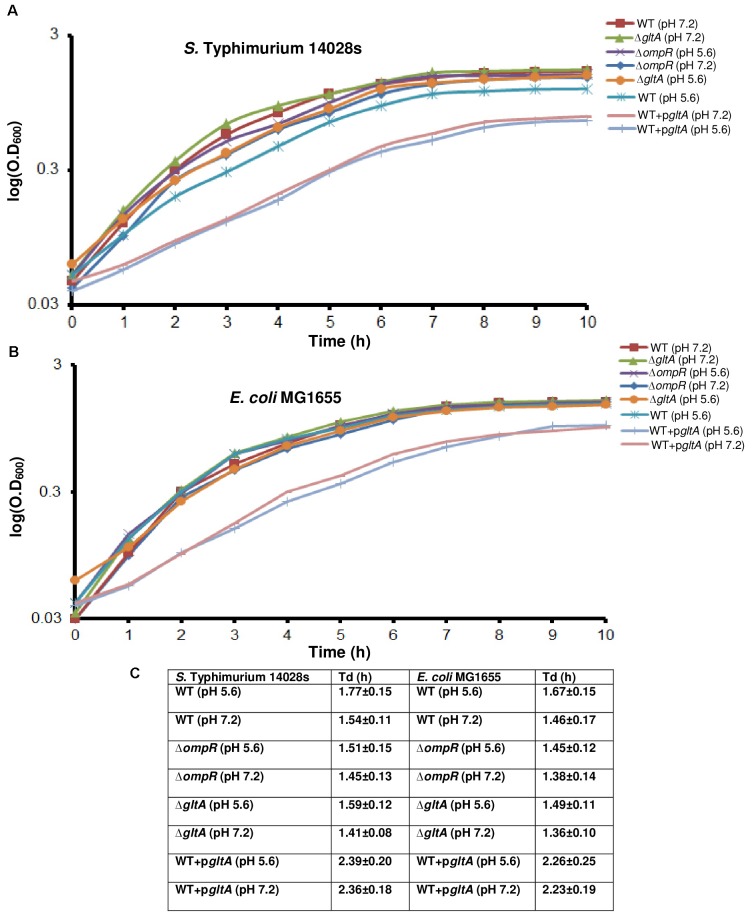
The effect of acid pH on growth curves of *S.* Typhimurium and *E. coli.* Wild-type, an *ompR* null mutant, a *gltA* null mutant and a *gltA* over-expressed strain of **(A)** Overnight cultures of *S.* Typhimurium and **(B)** *E. coli* grown in LB were sub-cultured (1:100) in MgM pH 7.2 for 24 h. The cultures were then sub-cultured again (1:50) in MgM pH 5.6 or pH 7.2 for an additional 10 h as described in Materials and Methods. The optical density at 600 nm (O.D._600_) was measured hourly to monitor bacterial growth (*n* = 3). Strains in which *gltA* was over-expressed exhibited growth defects at neutral and acid pH compared to the wild-type and *ompR* null strains in both *S.* Typhimurium and *E. coli*. The error bars were removed to make the graphs legible. **(C)** The doubling time (Td) was plotted as determined from the exponential curve fitting function as described in Materials and Methods. Error bars represent the mean ± standard deviation (*n* = 3).

We were interested in understanding the role of OmpR in the acid stress response. To address this, we first examined whether loss of OmpR led to a growth defect during acid stress. In Figure 1, the growth profiles of *S*. Typhimurium and *E. coli* wild-type and isogenic *ompR* null strains at neutral and acid pH were compared. At neutral pH, the growth of the wild-type and the *ompR* null strain of *S*. Typhimurium was nearly identical (Δ*ompR* Td = 94% of wild-type, Figure 1C). At acid pH, the growth of the *ompR* null strain was similar to the wild-type growth at neutral pH. However, the wild-type strain grew more slowly (Td = 87% of Td at neutral pH, Figure 1C) at acid pH (top panel). In *E. coli* the differences were similar to *S.* Typhimurium, although the Tds were all slightly faster (Figures 1B,C). Under these growth conditions, we previously measured the intracellular pH of wild-type *S.* Typhimurium (pH_i_ = 6.15) and *E. coli* (pH_i_ = 6.55) after 90 min exposure to pH_*e*_ 5.6 (see Supplementary Table S1). Thus, acid stress has only minor effects on *S*. Typhimurium and *E. coli* growth.

### Acid Stress Does Not Affect *ompR* Transcript Levels

**FIGURE 2:**
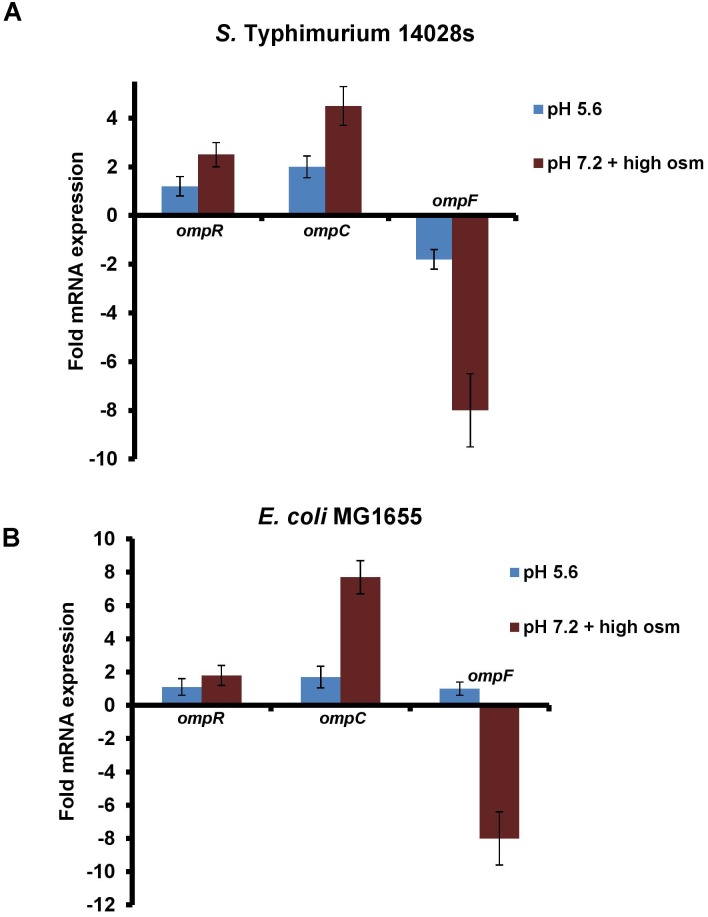
*ompR* transcription in *S*. Typhimurium and *E. coli* is insensitive to pH, but increases slightly at high osmolality. **(A)** The mRNA levels of *ompR*, *ompC* and *ompF* at pH_e_ 5.6 and pH_e_ 7.2 plus 15% (w/v) sucrose were compared to pH_e_ 7.2 by qRT-PCR in the wild-type *S*. Typhimurium and **(B)** *E. coli* strains. The mRNA expression level of the target genes was normalized relative to 16S rRNA. Error bars represent the mean ± standard deviation (*n* = 3).

Because cytoplasmic acidification during acid and osmotic stress was dependent upon OmpR, we first examined whether *ompR* was up-regulated during these stress conditions (Figure 2). We measured the *ompR* transcript levels by quantitative real-time polymerase chain reaction (qRT-PCR) in *S.* Typhimurium and *E. coli* after growth in acid or at high osmolality and compared *ompR, ompC* and *ompF* levels in the wild-type strains. The levels of *ompR* were insensitive to an acid pH shift, but increased at high osmolality in *S.* Typhimurium (2.3-fold) and in *E. coli* (1.8-fold) (Figure 2). The known *ompR*-regulated transcripts responded as predicted: *ompC* levels increased in response to acid pH, but were even higher at high osmolality, whereas *ompF* was repressed under acid stress compared to neutral pH. At high osmolality, *ompF* levels decreased in *S.* Typhimurium and *E. coli*, but *ompF* levels in *E. coli* were similar at acid and neutral pH (Figure 2). The observation that *ompR* transcripts did not change in acid pH was consistent with our previous studies, where we counted OmpR molecules by PALM imaging of an OmpR-PAmCherry photoactivatable fluorescent protein fusion. There was no difference in OmpR molecules in acidic conditions compared to neutral pH in *S.* Typhimurium (Liew et al., unpublished), and in *E. coli*, OmpR numbers were slightly lower at acid pH (Foo et al., 2015b) (see section “Discussion”).

### Identification of the OmpR Acid Stress Regulon

**FIGURE 3:**
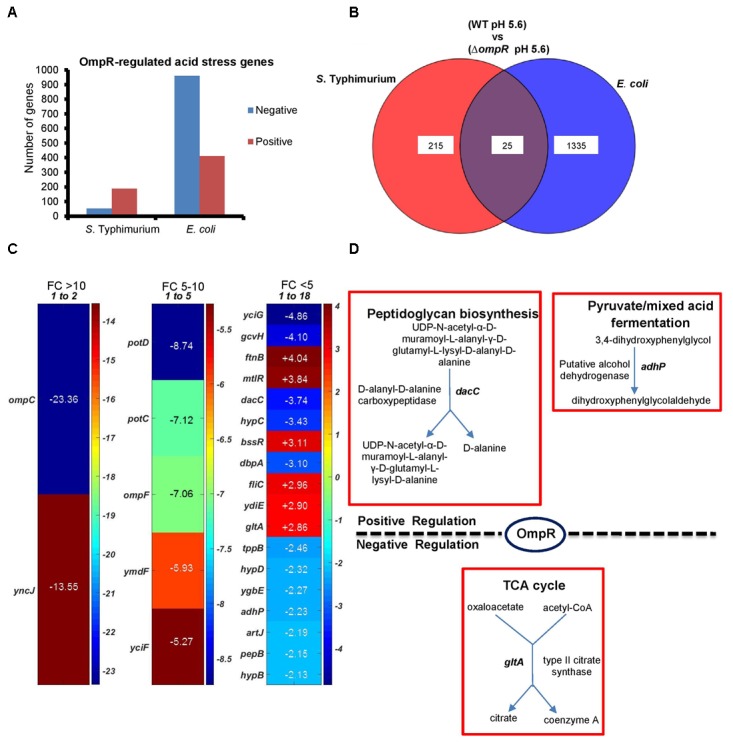
Comparison of the *ompR* acid stress regulons of *S*. Typhimurium and *E. coli* **(A)** The number of OmpR regulated genes (≥ 2-fold change; *P*-value ≤ 0.05) in *S*. Typhimurium and *E. coli* are shown in response to acid stress. **(B)** Overlap of OmpR acid stress targets between *S*. Typhimurium and *E. coli*. **(C)** A heat map shows the differential expression profiles of common OmpR targets separated into three groups based on the fold change, i.e., *ompC* was the most highly downregulated-regulated gene at –23.36. A positive number indicates OmpR repression. The fold change represents the expression level of an *ompR* null strain compared to the wild-type in *S*. Typhimurium or *E. coli* during acid stress. The color scale represents the average fold change (*n* = 3). Supplementary Table S2 provides a detailed description of the overlapping genes. Data processing was performed using MATLAB 6.1 (MathWorks Inc., Natick, MA, United States). **(D)** Enzymatic reactions and pathways of the common OmpR targets involved in metabolism are shown using PGDB analysis (*EcoSal Plus* doi: 10.1128/ecosalplus.ESP-0009-2014). Similar algorithms and software tools were used for the remaining microarray analyses.

In order to identify OmpR-dependent pathways induced during acid stress, gene expression profiles between wild-type and an *ompR* null strain of *S*. Typhimurium and *E. coli* were compared. Genes with statistically significant differential expression were required to meet two criteria: a fold change (FC) ≥ 2 and a *P*-value of ≤ 0.05, as determined by the Student’s *t*-test (Figure 3). The OmpR acid stress regulon was considerably more extensive in *E. coli* compared to *S*. Typhimurium, where a higher number of OmpR affected genes were apparent (1360 vs. 240). In *S*. Typhimurium, more acid-sensitive genes (78%) were positively regulated by OmpR (Figure 3A), i.e., they were down-regulated when OmpR was absent. In contrast, *E. coli* was poised in the opposite direction, i.e., OmpR repressed the majority of the acid-responsive genes (71%). As shown in Figure 3B, it was evident that there was significant divergence between *S*. Typhimurium and *E. coli* OmpR acid stress regulons, as only 25 targets were common to both organisms. These 25 genes represented <2% of the overall OmpR response in *E. coli*, but were ∼10% of the *S.* Typhimurium OmpR-dependent acid stress response (Figures 3B,C).

We used the UniProt Gene Ontology (GO) annotation program to represent the normal molecular function and biological processes of the 25 common targets (Supplementary Table S2A). As intracellular acidification in wild-type *S*. Typhimurium and *E. coli* did not result in significant growth defects (Figure 1), we searched for biosynthetic and metabolic pathways and/or for pathways that might prevent accumulation of metabolites or toxins in order to support growth at acid pH. To gain insight into the metabolic functions, significantly differentiated genes were matched with the BioCyc Database collection (PGDB) (Keseler et al., 2011). The OmpR overlapping targets revealed three genes (*dacC, gltA and adhP*) involved in metabolic pathways (Figure 3D). DacC is a D-alanyl-D-alanine carboxypeptidase that was activated by OmpR (3.7-fold) (see section “Discussion”). OmpR down-regulated the citrate synthase *gltA* (2.9-fold), which synthesizes citrate from oxaloacetate and acetyl-CoA. A previous report showed that the expression of *gltA* was inversely proportional to the cell growth rate in *E. coli* (Park et al., 1994). In another study *S*. Typhimurium *gltA* was shown to catalyze the accumulation of 2-methylcitrate, which is deleterious to cell growth (Horswill et al., 2001). In both *S*. Typhimurium and *E. coli*, over-expression of *gltA* resulted in growth defects (Figures 1A,B), as evident by the increased doubling times (Td) at acidic (∼25%) and neutral pH (∼34%) (Figure 1C). Thus, OmpR repression of *gltA* contributes to optimum growth when *S*. Typhimurium and *E. coli* are acidified. A systems biology approach also identified *gltA* as being down-regulated in response to acid stress (Stincone et al., 2011). Cluster analysis of the common targets in *S*. Typhimurium and *E. coli* identified shared functions such as membrane transport (*tppB, ompC, ompR, potC*, *potD*, *ygbE*), biosynthesis of antibiotics and secondary metabolites (*adhP*, *gltA*, *gcvH*, *dacC*, *pepB*) and others (see Supplementary Table S2B for the full list).

### Analysis of the OmpR Osmotic Stress Regulon

**FIGURE 4:**
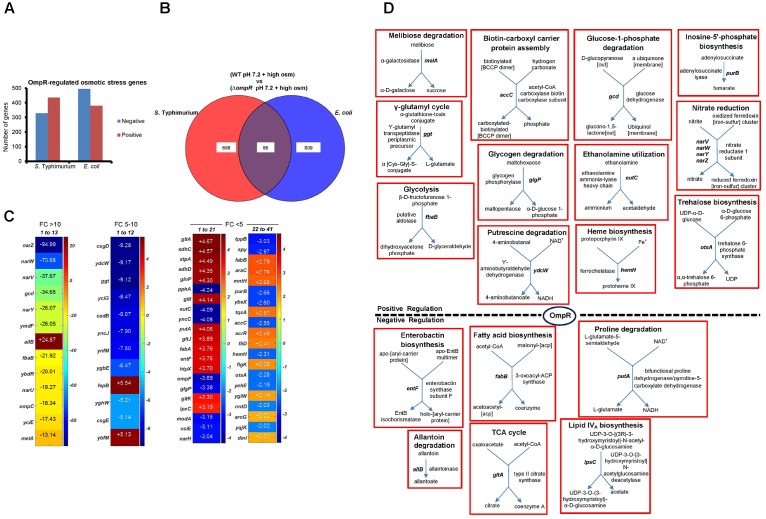
Analysis of OmpR osmotic stress-sensitive targets in *S*. Typhimurium and *E. coli*. **(A)** The number of differentially expressed (≥ 2-fold change; *P*-value ≤ 0.05) OmpR-affected genes in *S*. Typhimurium and *E. coli* are indicated. **(B)** Overlapping OmpR targets between *S*. Typhimurium and *E. coli* during osmotic stress are shown. **(C)** A heat map representing the expression profiles of the overlapping OmpR targets are grouped based on their fold change. The color scale represents the average fold change of *ompR* null strain compared to the wild-type in *S*. Typhimurium (*n* = 3). Supplementary Table S3 details a description of the overlapping genes. **(D)** Metabolic maps of overlapping OmpR targets involved in cellular processes are shown.

In repsonse to osmotic stress, the cytoplasm of both *E. coli* and *S*. Typhimurium was acidified in an OmpR-dependent manner. However, acidification was not as pronounced as during acid stress and the pathways involved were distinct (Chakraborty et al., 2015, 2017). We therefore examined the OmpR regulon in response to osmotic stress. At high osmolality, the number of OmpR-regulated genes was similar between *E. coli* (875) and *S*. Typhimurium (764) (Figure 4A). Sixty six OmpR targets that were sensitive to changes in osmolality were overlapping, comprising ∼8–9% of the total OmpR response in either *S*. Typhimurium or *E. coli* (Figure 4B).

Uniprot GO was used to list the functions of the 66 common OmpR targets (Supplementary Table S3A). Interestingly, genes encoding nitrate reductase such as *narZ* (95-fold), *narW* (71-fold) and *narV* (38-fold) showed the highest decrease in the *ompR* null strain compared to the wild-type, indicating that they were strongly activated (directly or indirectly) by OmpR (Figure 4C). Growth inhibition by accumulation of nitrate during osmotic stress has been reported in the sulfate-reducing bacterium *Desulfovibrio vulgaris* (He et al., 2010). Thus, up-regulation of nitrate reductase is a possible mechanism employed by wild-type *S*. Typhimurium and *E. coli* to relieve nitrate toxicity at high osmolality. PGDB analysis of the overlapping targets identified 18 genes involved in various metabolic functions (Figure 4D). Cluster analysis identified eight major clusters linked to biological processes, including: nitrate metabolism, membrane transport, biosynthesis of metabolites and amino acids and transcriptional regulation (Supplementary Table S3B).

### Common OmpR Targets in Acid and Osmotic Stress

**FIGURE 5:**
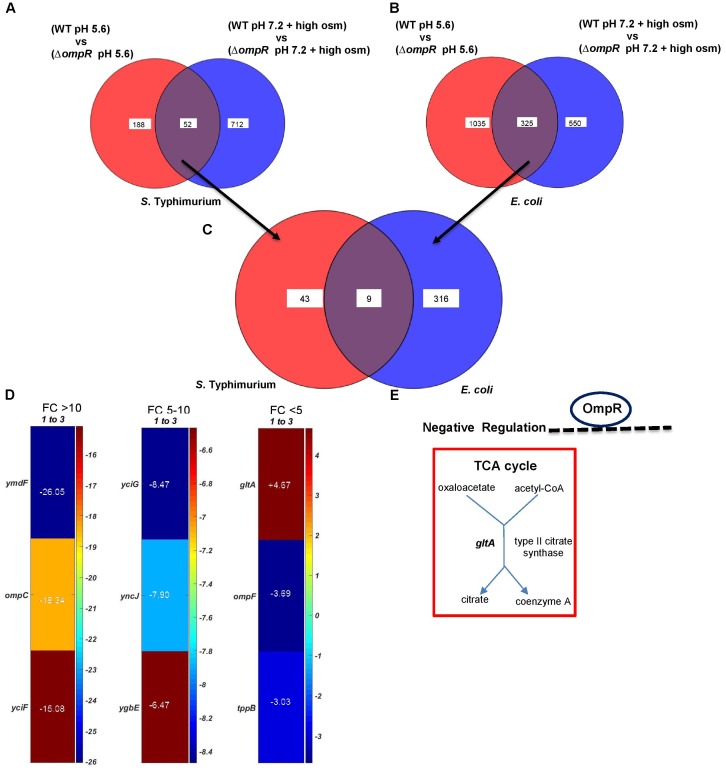
Overlapping OmpR acid and osmotic stress targets. Common OmpR targets in **(A)** *S*. Typhimurium and **(B)** *E. coli* responsive to both acid and osmotic stress are shown. **(C)** Overlapping genes between **(A)** and **(B)** identifies nine common OmpR targets differentially expressed during both pH and osmolality shifts. **(D)** A heat map shows the expression profiles of the nine common OmpR targets. The color scale represents the average fold change of an *ompR* null strain of *S*. Typhimurium compared to the wild-type (*n* = 3). Supplementary Table S4 provides a detailed description of the overlapping genes. **(E)** A metabolic map of *gltA* is shown.

We next identified the OmpR targets that were involved in response to both acid and osmotic stress. In *S*. Typhimurium, 52 genes were common OmpR targets responsive to both stress pathways (Figure 5A), and in *E. coli*, 325 genes were common (Figure 5B). Out of these, nine genes were common to the OmpR regulons of *S*. Typhimurium and *E. coli* (Figure 5C). Three of the nine genes were again the well-known OmpR targets, *ompC*, *ompF* and *tppB* (Goh et al., 2004). Five genes were of unknown function (Figure 5D and Supplementary Table S4A). These nine genes were grouped into the membrane transport cluster (Supplementary Table S4B).

**FIGURE 6:**
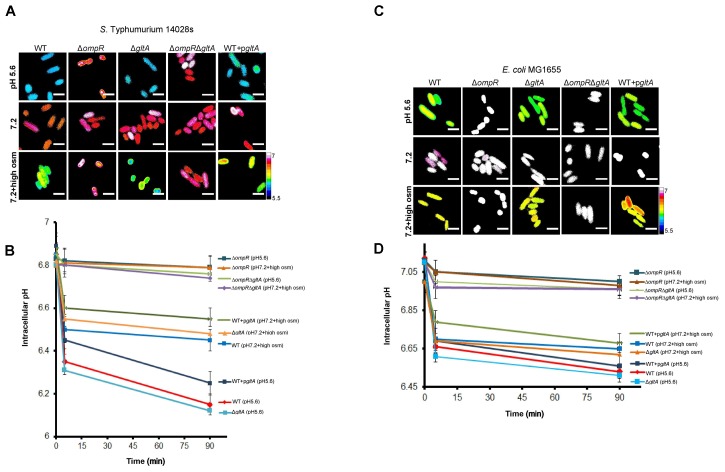
GltA is not involved in intracellular acidification. *S*. Typhimurium and *E. coli* cultures were incubated with 20 μM BCECF for 60 min before imaging. Representative epifluorescence ratio images (R488/440) of emission channel 525 nm upon 488 and 440 nm excitation were obtained for wild type, an *ompR* null mutant, a *gltA* null mutant, an *ompR*/*gltA* null strain and a *gltA* over-expressed strain of **(A)** *S*. Typhimurium and **(C)** *E. coli* incubated at either acid pHe (5.6), pHe (7.2), or pHe (7.2) plus 15% (w/v) sucrose. Using ImageJ software, ratio images were color coded blue (ratio = 0.1) to white (ratio = 1). Scale bar, 3 μm. A plot of the intracellular pH of 50 cells of wild-type and mutants of **(B)** *S*. Typhimurium and **(D)** *E. coli* at each indicated time point. Error bars represent the mean ± s.e.m. (*n* = 3).

PGDB analysis identified *gltA* as the sole OmpR target involved in metabolism (Figure 5E). Thus, OmpR repression of *gltA* appears to play a major role in supressing growth defects upon intracellular acidification in wild-type *S*. Typhimurium and *E. coli* in response to both acid and osmotic stress. To determine if *gltA* was involved in intracellular acidification, we measured the pH_i_ of *gltA* null strains and strains of *S*. Typhimurium and *E. coli* in which *gltA* was over-expressed in response to both acid and osmotic stress. The *gltA* null strains were fully capable of cytoplasmic acidification and the pH_i_ was similar to the wild-type in both *S*. Typhimurium and *E. coli* (Figures 6A–D). Similarly, in the Δ*ompR/gltA* null strains, the pH was similar to the pH of the *ompR* null strain. This was not surprising, because citrate synthase (*gltA*) is not known to be involved in proton exchange. In contrast, the *gltA* over-expressed strains exhibited a slightly higher intracellular pH (∼0.1 pH unit) compared to the wild-type strains.

**FIGURE 7:**
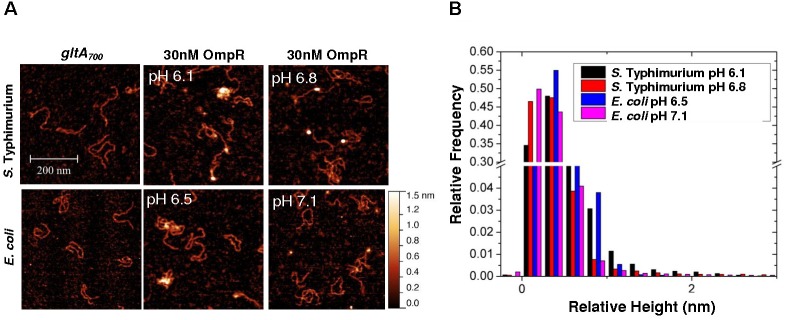
OmpR binds to the *gltA* promoter. **(A)** AFM images of the 700 bp *gltA* promoter (*gltA_700_*) from *S*. Typhimurium and *E. coli* (left panel) with 30 nM OmpR at either acidic (middle panel) or neutral pH (right panel). The pH corresponds to the relevant pH_i_ that was measured during acid stress (Chakraborty et al., 2015, 2017). **(B)** A relative height distribution histogram of the *gltA* promoter complexed with 30 nM OmpR at either acid or neutral pH. To obtain the relative height distribution histograms of OmpR-*gltA* complexes, a threshold was applied to filter the *gltA* contour from the background by a MATLAB code. The relative height, which is essentially the pixel values of the contour above the background, was plotted as a distribution histogram. A higher relative height indicates more OmpR bound to the DNA. The relative frequency indicates how often the relative height was observed in acid or neutal pH. The term relative height is used, as the apparent heights measured by AFM do not represent the true height (Lai et al., 2015). In the absence of DNA, OmpR was visible as pinpoint dots and was not aggregated (Supplementary Figure S1).

To determine whether *gltA* repression was the result of direct interaction by OmpR, we used AFM to visualize OmpR binding to P*gltA*. OmpR was added to a solution buffered to the measured pH_i_ of *S*. Typhimurium or *E. coli*, as determined from previous experiments (Chakraborty et al., 2017). Addition of OmpR at pH 6.1 (*S*. Typhimurium) or at pH 6.5 (*E. coli*) increased the proportion of DNA-OmpR protein complexes (Figure 7A), as evident by an increase in the relative height (white foci). OmpR binding to the *gltA* promoter was increased at acid pH ompared to the addition of OmpR at neutral pH (Figures 7A,B). Thus, OmpR represses *gltA* during acidification via a direct interaction at its promoter to enable optimum growth in both *S*. Typhimurium and *E. coli*. In previous studies, we also determined that there was no visible effect of acid pH on OmpR (pH 6.1–7.1) in the absence of DNA (Chakraborty et al., 2017) (see Supplementary Figure S1).

### Comparison of Osmolytes in the OmpR *S*. Typhimurium Transcriptome- Salt *vs.* Sucrose

**FIGURE 8:**
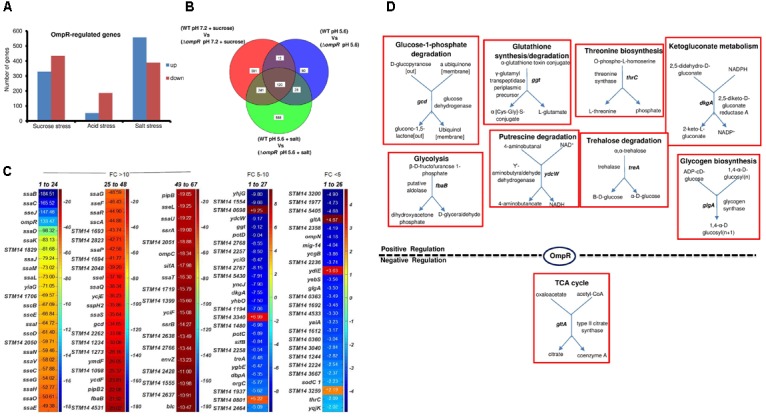
Overlapping OmpR acid, sucrose, and salt stress targets **(A)** Statistically significant (≥ 2-fold change; *P*-value ≤ 0.05) OmpR targets differentially expressed in *S*. Typhimurium in response to acid, sucrose and salt stress are shown. **(B)** Overlapping OmpR targets are shown. **(C)** A heat map shows the expression profiles of the overlapping OmpR-regulated genes grouped based on differences in their fold change. The color scale represents the average fold change of an *ompR* null strain during sucrose stress (*n* = 3). **(D)** Metabolic overview of OmpR targets involved in cellular processes is shown.

Our previous data showed that the local unfolding and ensemble behavior of EnvZc was comparable in the presence of either sucrose or salt as the osmolyte (Wang et al., 2012). We were curious whether there were separate effects of osmolytes depending on whether the osmolyte was ionic or non-ionic. We therefore analyzed the effect of salt by adding 400 mM NaCl to N-minimal medium (MgM) at pH_e_ 5.6 (976 mOsmol/kg). We compared OmpR-dependent pathways induced by acid, sucrose and salt stress from the expression profiles of wild-type and the *ompR* null strain of *S*. Typhimurium (Figure 8). Salt stress resulted in the highest number of differentially expressed genes (947) compared to acid (240) and sucrose (764) stress (Figure 8A). There was moderate overlap of these three responses, as 120 genes were common OmpR targets, but >50% of these were uncharacterized genes. This core regulon represented ∼13% of the OmpR response to salt stress (Figures 8B,C). It was noteworthy that half of the most highly expressed genes (fold change >10) were SPI-2 or SPI-2 co-regulated genes, which enable *S*. Typhimurium to survive the high osmolality and low pH of the macrophage vacuole (Chakraborty et al., 2015). Apart from *ssrA* and *ssrB*, none of the other SPI-2 genes have been shown to be direct targets of OmpR regulation (Lee et al., 2000; Feng et al., 2003, 2004), although many of them are directly regulated by SsrB (Feng et al., 2004; Walthers et al., 2007, 2011). PGDB analysis of the 120 OmpR common targets revealed only nine genes involved in metabolism (Figure 8D), and most of these were degradative pathways. Interestingly, *gltA* was the only OmpR-repressed target that was sensitive to acid, sucrose and salt stress in *S*. Typhimurium (Figure 8D) and in *E. coli* during acid and sucrose stress (Figure 5D). Our findings suggest that in both *S.* Typhimurium, *S.* Typhi (Perkins et al., 2013) and *E. coli*, OmpR repression of *gltA* plays a major role in maintaining optimum cell growth during stress.

## Discussion

### OmpR Is an Important Global Regulator of the Bacterial Response to Acid/Osmotic Stress

Bacteria encounter diverse environmental conditions both inside and outside of the host. TCRSs play a major role in sensing these varied environmental cues and subsequently modulate gene expression in response to stress. In a previous study, we established that the cytoplasmic domain alone of the sensor kinase EnvZ sensed cytoplasmic signals to activate its downstream target OmpR without being in the membrane (Wang et al., 2012). It was then logical that **cytoplasmic** acidification of *S*. Typhimurium occurred during macrophage infection (Chakraborty et al., 2015) and during *in vitro* acid and osmotic stress (Chakraborty et al., 2017). Cytoplasmic acidification was completely dependent on the OmpR response regulator, but did not require known OmpR-regulated genes such as *ompC*, *ompF*, or *ssaC* (a SPI-2 structural gene). To elucidate the OmpR regulatory networks, we performed microarray analysis and compared the transcriptome of *ompR* null and wild-type strains of *S*. Typhimurium and *E. coli* during acid and osmotic stress. OmpR repressed distinct genes, depending on whether the stressor was acid or osmotic stress, as driven by differences in pH_i_ that ensued (Chakraborty et al., 2017). Thus, we were motivated to further understand the role of OmpR in the cellular stress response.

### OmpR Down-Regulates a Metabolic Pathway That Produces Toxic Intermediates

A previous study proposed a program of gene expression of *E. coli* BW35113 during exposure to acid pH that involved the following metabolic switches: from utilizing glucose to gluconeogenesis and fatty acid synthesis, from aerobic to anaerobic growth and down–regulation of fumarate and up-regulation of formate and nitrate pathways (Stincone et al., 2011). In the present work, some of these same changes were evident in the response to acidification caused by osmotic stress, where *nar* (*ZWVY* and *U*) genes were highly up-regulated (Figure 4C), but fatty acid synthesis genes (*entF, fabB* and *lpxC*) were down-regulated. Common targets between *E. coli* and *S.* Typhimurium also included genes involved in carbohydrate degradation (*melA*, *gcd, ydcW, fbaB, and glgP*).

During acid stress, the OmpR overlapping targets revealed three genes in both *E. coli* and *S.* Typhimurium involved in metabolism, including: *dacC, gltA and adhP* (Figure 3D). DacC is a D-alanyl-D-alanine carboxypeptidase that was activated by OmpR (3.7-fold). Deletion of DacC (PBP6) had no detectible phenotype in *E. coli* (Broome-Smith and Spratt, 1982), but over-expression was reported to be toxic (Pedersen et al., 1998). In another study, *dacC* was shown to be essential for *E. coli* cell morphology and was regulated by BolA (Santos et al., 2002).

Nine common targets in the OmpR-dependent acid and osmotic stress response were identified. Three of these were *ompF, ompC* and *tppB*, i.e., known OmpR-regulated genes. The fourth was *gltA*, which encodes citrate synthase, and converts oxaloacetate and acetyl-CoA into citrate (Figures 3–6). GltA controls the entry of metabolites into the TCA cycle, and it also appears to be the source of a toxic metabolite, 2-methylcitrate that can be a potent inhibitor of cell growth (Horswill et al., 2001). The *gltA* promoter is regulated by ArcA (Park et al., 1994) and in the present work we establish that OmpR binds to P*gltA* and represses its expression to ensure optimum growth. Over-expression of *gltA* resulted in growth defects in both *S*. Typhimurium and *E. coli* (Figures 1, 7). OmpR regulation of *gltA* also appeared in a ChIP-seq analysis of *S.* Typhi (Perkins et al., 2013). A clearer picture will hopefully emerge when we understand the function of these unknown genes.

Although the remaining five common OmpR target genes are of unknown function, they include YmdF and YciG, annotated as stress-induced proteins related to conidiation-specific protein 10 from *Neurospora*. YciF is highly conserved across the *Enterobacteriaciae*, and based on its structure, it was proposed to be a metal binding protein (Hindupur et al., 2006), and to function to protect the cell against oxidative damage. YciF was up-regulated in the OmpR response to acidification by acid or osmotic stress (Figure 5). It is annotated as being involved in the cellular response to DNA damage, as is *yciG*. YciF and YciG are in the *yciFGE-katN* operon, KatN is a non-heme catalase (Robbe-Saule et al., 2001). In *Salmonella*, YciF was reported to be under positive control of RpoS (Ibanez-Ruiz et al., 2000), yet our previous study showed that OmpR repressed *rpoS* during osmotic stress (Chakraborty et al., 2017), which would decrease *yciF* transcription. Our microarray results identify *yciF* as up-regulated by OmpR in both osmotic and acid stress (Figure 5). In *Salmonella*, *yciF* was reported to be regulated by bile, independently of *rpoS* (Prouty et al., 2004).

### The *E. coli* Acid Stress Regulon Is Large

Our microarray results indicated that the OmpR-dependent response to acid stress in *E. coli* involved about six times as many genes as in *S*. Typhimurium (Figure 3). The involvement of 1360 genes in the OmpR-dependent *E. coli* MG1655 acid stress response (and 1538 genes in the total OmpR-independent acid stress response) was similar to a study of *E. coli* BW25113, in which 1871 genes were differentially expressed after a 15 min acid exposure (to pH 5.5) (Stincone et al., 2011). These two studies were in contrast to a ChIP-on-chip study using the *E. coli* K-12 strain CSH-50 (Quinn et al., 2014). In that study, only 144 OmpR-regulated acid stress genes were identified in CSH-50, i.e., only ∼10% compared to the 1360 genes that we identified in *E. coli* MG1655. In contrast, the response of *S*. Typhimurium was comparable between our study and the study by Quinn and colleagues: 240 vs. 212 acid stress genes (Figure 3; Quinn et al., 2014, respectively). Fifteen OmpR-regulated genes were common to *E. coli* CSH-50 and *S.* Typhimurium (Quinn et al., 2014), whereas in *E. coli* MG1655 and *S.* Typhimurium strain 14028, 25 genes were common (Figure 3). There was very little overlap between the common genes that we identified and the common genes identified by Dorman and co-workers (Quinn et al., 2014); although the few genes that were in common were again the known OmpR targets: *ompC*, *ompF* and the tripartite permease, *tppB*. These same targets appear in the common genes of the acid and osmotic stress responses of *S.* Typhimurium and *E. coli* (see Figure 5). CSH-50 is a proline and thiamine auxotroph, contains an insertion sequence in *fimE*, and is *rpsL* null (Miller, 1972; Blomfield et al., 1991). Thus, extensive genetic differences between *E. coli* K-12 CSH-50 and MG1655 likely explains the poor overlap between studies.

### OmpR Numbers and pH Regulation

Our qRT-PCR results are in good agreement with our microarray; the *ompR* transcripts of both *S.* Typhimurium and *E. coli* were unchanged in acid pH, but were slightly up-regulated by ∼2-fold in response to osmotic stress (Figure 2). This result conflicts with a previous study that reported *ompR* transcripts in *S*. Typhimurium were ∼2.7-fold higher at pH 4.5 compared to pH 7 (Quinn et al., 2014). It may be that the lower pH examined (4.5 instead of 5.6), or differences in the culture media (EG-minimal medium instead of MGM) or the reference gene employed (*gmk*) might contribute to these discrepancies. However, our finding that *ompR* transcripts did not change in acid stress (at pH 5.6) was entirely consistent with our use of super-resolution imaging to count OmpR molecules (Foo et al., 2015b; Liew et al., unpublished). OmpR molecules were counted in acid and neutral pH and the number of OmpR molecules was similar. We then used single particle tracking photoactivation localization microscopy (Spt-PALM) to monitor OmpR binding to DNA by measuring diffusion coefficients in acid and neutral pH. OmpR binding only increased by 5% in acid pH (Liew et al., unpublished). Previously, we have used AFM extensively to examine the role of pH in OmpR binding to DNA. OmpR binding affinity at the *ompC* and *cadB/A* promoters was increased in acid compared to neutral pH (Chakraborty et al., 2017). We propose that rather than increasing the number of OmpR molecules in acid pH, the OmpR binding affinity for DNA is pH-sensitive and increases in acid pH. In *E. coli* BW25113, it was reported that *ompR* transcripts were reduced by ∼50% in acid pH (Stincone et al., 2011). This was surprising, given the substantial role that OmpR plays in the acid stress response. In contrast, in the CSH-50 *E. coli* strain, *ompR* transcripts were unchanged between pH 7 and pH 4.5 (Quinn et al., 2014).

### What Is the Cellular Response to Acid pH?

It was suggested that DNA topological changes at acid pH could drive OmpR binding to DNA and might be responsible for an increase in the number of OmpR-bound genes observed during acid stress (Cameron and Dorman, 2012). Previous studies used OmpR-dependent transcriptional fusions to *gfp* (including: *ompR-gfp* and *ssrA-gfp*) and reported that transcription was increased in the presence of novobiocin, which presumably reduces supercoiling. The authors concluded that DNA relaxation promoted OmpR binding to DNA, enhancing transcription (Cameron and Dorman, 2012). This was surprising, because we required supercoiled templates for OmpR transcription *in vitro* (D. Walthers and L.J. Kenney, unpublished observations). We used super-resolution microscopy to image a chromosomally-encoded OmpR-PAmCherry fusion protein during osmotic and acid stress (Foo et al., 2015b). We examined both the OmpR distribution and chromosomal compaction and discovered that the chromosome was actually more condensed during acidic conditions, rather than being more relaxed (Foo et al., 2015b; Liew et al., unpublished). This finding was recapitulated in a recent study of the nucleoid-associated protein H-NS (Gao et al., 2017).

### A Caution Regarding C-Terminal Fusions to OmpR

ChIP-on-chip results with OmpR showed increased binding of OmpR at the *mgtC* promoter (Quinn et al., 2014), although microarray and qRT-PCR analysis of OmpR-regulated genes at acid pH did not identify *mgtC* as a target of OmpR regulation, nor did *mgtC* contribute to intracellular acidification (Chakraborty et al., 2015). A likely explanation for this discrepancy is that OmpR containing a 3XFLAG tag was employed in the ChIP-on-chip study, which also contained a D55E substitution (Quinn et al., 2014). It is well known that C-terminal tags to OmpR affect not only its DNA binding ability (Perkins et al., 2013), but also its specificity (Shimada et al., 2015), which may explain why it was observed that OmpR preferred relaxed DNA over supercoiled DNA. The D55E-3XFLAG-tagged OmpR also required concentrations > 1 μM to bind in electrophoretic mobility shift assays (Cameron and Dorman, 2012), even though the affinity of wildtype, unphosphorylated OmpR for the porin genes *ompF* and *ompC* is ∼150 nM (Head et al., 1998). In a recent study, OmpR targets were identified by SELEX (Shimada et al., 2015), but we were unable to validate the targets identified (Gao and Kenney, unpublished observations). Furthermore, different OmpR targets were identified depending on whether a C-terminal or N-terminal tag was employed (Shimada et al., 2015). Thus, extreme caution should be used when interpreting results using OmpR C-terminal fusions. A more likely explanation is that OmpR directly regulates *phoP*, in agreement with (Quinn et al., 2014), where OmpR was shown to bind to the *phoP* promoter. In turn, PhoP directly regulates *mgtC* and the reduced *mgtC* levels observed in the *ompR* null strain were likely due to reduced *phoP* levels. This type of indirect regulation has been observed in the OmpR regulation of the response regulator SsrB, which then regulates *sifA* (Walthers et al., 2011). It is also evident in the additional SPI-2 genes that are known SsrB targets (Figure 8).

Overall, this work revealed a large number of genes that are new targets of OmpR regulation during acid and osmotic stress. The challenge will be to determine whether these are direct effects or if they are mediated through OmpR regulation of an intermediating regulator, as we observed with the OmpR repression of the stationary phase sigma factor *rpoS* (Chakraborty et al., 2017).

## Author Contributions

SC performed the analysis and the experiments. SC and LK analyzed the data and wrote the manuscript.

## Conflict of Interest Statement

The authors declare that the research was conducted in the absence of any commercial or financial relationships that could be construed as a potential conflict of interest.

## Footnotes

1 http://gwyddion.net/

## Abbreviations

BCECF-AM: 2′,7′-Bis-(2-Carboxyethyl)-5-(and-6)Carboxyfluorescein, AcetoxymethylEster)
CAD: cadaverine decarboxylation system
cF: 5 (and 6-)-carboxyfluorescein
FRET: fluorescence resonance energy transfer
GAD: glutamine decarboxylation system
min: minutes
pH_e_: extracellular pH
pH_i_: intracellular pH
qRT-PCR: quantitative real-time polymerase chain reaction
RT: room temperature
SPI-2: *S*. Typhimurium pathogenicity island 2
Spt-PALM: single particle tracking photoactivatable localization microscopy
TCRS: two-component regulatory systems
TTSS: type three secretion systems.
