# Supplementary material for: A New Role of OmpR in Acid and Osmotic Stress in Salmonella and E. coli
Source: Front Microbiol. 2018 Nov 22;9:2656. doi: 10.3389/fmicb.2018.02656 (PMC6262077; doi:10.3389/fmicb.2018.02656)
Supplement: Supplementary file 1 [file Data_Sheet_1.PDF]

frontiers

Supplementary Material

A New Role of OmpR in Acid and Osmotic Stress in *Salmonella* and *E. coli*

Smarajit Chakraborty and Linda J. Kenney*

*Author for correspondence: kenneyl@uic.edu

Supplementary Data: Supplementary Tables S1-S5 and Supplementary Figure 1

TABLES

Table S1. Intracellular pH values in response to acid and osmotic stress

S. Typhimurium						
Time (min)	WT (pH 5.6)	WT (pH 7.2)	Δ*ompR* (pH 5 .6)	Δ*ompR* (pH 7.2)	WT (pH 7.2 high osm)	Δ*ompR* (pH 7.2 high osm)
0	6.80	6.80	6.83	6.80	6.85	6.83
5	6.35	6.82	6.80	6.81	6.51	6.82
90	6.15	6.80	6.75	6.75	6.45	6.75
E. coli						
Time	WT (pH 5.6)	WT (pH 7.2)	Δ*ompR* (pH 5.6)	Δ*ompR* (pH 7.2)	WT (pH 7.2 high osm)	Δ*ompR* (pH 7.2 high osm)
0	7.13	7.13	7.10	7.10	7.13	7.13
5	6.65	7.10	7.08	7.13	6.79	7.10
90	6.55	7.13	7.05	7.10	6.75	7.10

Table S2A. Description of the overlapping OmpR targets in response to acid stress

No	Gene	Protein	Molecular process	Biological process	FC	Regulation
1	*ompC*	Outer membrane protein C	Metal ion binding, Porin activity	Ion transport, Cellule response to DNA damage	23.36	down
2	*yncJ*	Uncharacterized GST-like protein YncJ	Glutathione Transferase activity	Glutathione Metabolism	13.54	down
3	potD	Spermidine/putrescine-binding periplasmic protein	polyamine-transporting ATPase activity	Polyamine transport	8.74	down
4	*potC*	Spermidine/putrescine-transport system permease protein	Polyamine-transporting ATPase activity	Polyamine transport	7.12	down
5	*ompF*	Outer membrane protein F	Drug transmembrane transporter activity, Porin activity	Ion transport, drug transmembrane transport	7.05	down
6	*ymdF*	Uncharacterized protein YmdF	Uncharacterized	Uncharacterized	5.93	down
7	*yciF*	YciF	Uncharacterized	Cellular response to DNA damage stimulus	5.26	down
8	*yciG*	Uncharacterized protein YciG	Uncharacterized	bacterial-type flagellum-dependent swarming motility	4.85	down
9	*gcvH*	Glycine cleavage system H protein	lipoic acid binding	glycine decarboxylation via glycine cleavage system	4.10	down
10	*ftnB*	Bacterial non-heme ferritin-like protein	ferric iron binding	intracellular sequestering of iron ion	4.04	up
11	*mtlR*	Mannitol operon repressor protein MtlR	DNA binding	negative regulation of transcription	3.83	up
12	*dacC*	D-alanyl-D-alanine carboxypeptidase DacC	carboxypeptidase activity, penicillin binding	cell wall organization, peptidoglycan biosynthetic process	3.74	down
13	*hypC*	Hydrogenase isoenzymes formation protein HypC	iron ion binding	protein maturation	3.43	down
14	*bssR*	Biofilm regulator BssR	global regulator of several genes	regulation of gene expression	3.11	up
15	*dbpA*	ATP-dependent RNA helicase DbpA	ADP binding, ATPase activity	ribosomal large subunit assembly, RNA secondary structure unwinding	3.10	down
16	*fliC*	Flagellin protein FliC	structural molecule activity	bacterial-type flagellum-dependent cell motility	2.96	up
17	*ydiE*	Uncharacterized protein YdiE	Uncharacterized	Uncharacterized	2.90	up
18	*gltA*	Citrate synthase Protein GltA	citrate (Si)-synthase activity	tricarboxylic acid cycle	2.86	up
19	*tppB*	Dipeptide and tripeptide	antibiotic transporter activity, dipeptide	antibiotic transport, hydrogen ion transmembrane transport	2.46	down

		permease A	transmembrane transporter activity, symporter activity			
20	*hypD*	Hydrogenase isoenzymes formation protein HypD	4 iron, 4 sulfur cluster binding	cellular protein modification process	2.31	down
21	*ygbE*	Inner membrane protein YgbE	Uncharacterized	Uncharacterized	2.27	down
22	*adhP*	Alcohol dehydrogenase, propanol-preferring protein AdhP	alcohol dehydrogenase (NAD) activity	acetaldehyde catabolic process, response to ethanol	2.22	down
23	*artJ*	ABC transporter arginine-binding protein 1	amino acid binding, arginine binding	arginine transport, L-arginine import into cell	2.18	down
24	*pepB*	Peptidase B	aminopeptidase activity, manganese ion binding	peptide catabolic process	2.15	down
25	*hypB*	Hydrogenase isoenzymes nickel incorporation protein HypB	GTPase activity, nickel cation binding	cofactor biosynthetic process, protein complex assembly	2.12	down

Table 2.

Table S2B. Cluster analysis of overlapping OmpR targets upon acid stress

**Cluster 1**					
Category	Term	Count	P_value	Benjamini	Genes
KEGG Pathway	Biosynthesis of antibiotics	3	3.5E-1	9.6E-1	*adhP, gltA, gcvH, dacC, pepB*
KEGG Pathway	Biosynthesis of Secondary metabolites	3	5.7E-1	9.9E1	
KEGG Pathway	Metabolic pathways	5	7.0E-1	1.0E-0	
**Cluster 2**					
Category	Term	Count	P_value	Benjamini	Genes
Up Keywords	Transport	5	2.1E-1	9.9E-1	*tppB, ompC, ompF, potC, potD, ygbE,*
Up Keywords	Transmembrane	5	8.2E-1	1.0E-0	
Up Keywords	Membrane	5	8.9E-1	1.0E-0	
Up Keywords	Transmembrane helix	3	9.8E-1	1.0E-0	
**Non-Cluster**					
Category	Term	Count	P_value	Benjamini	Genes
Up Keywords	Signal	6	9.4E-2	9.7E-1	*ompC, ompF, artJ,potD, yncJ, dacC, fliC, potC, pepB, dbpA, gltA, ftnB, adhP,*
Up Seq Feature	Signal peptide	6	1.3E-1	8.8E-1	
KEGG Pathway	Two-component system	3	2.6E-1	1.0E-0	
KEGG Pathway	ABC transporters	3	2.8E-1	9.8E1	
GOTERM CC Direct	Cytoplasm	4	6.6E-1	1.0E-0	
Category	Term	Count	P_value	Benjamini	
Up Keywords	Hydrolase	3	6.7E-1	1.0E-0	
Up Keywords	Metal-binding	3	7.2E-1	1.0E-0	
Up Keywords	Cytoplasm	3	7.7E-1	1.0E-0	

Table S3A. Description of overlapping OmpR in response to osmotic stress

No	Gene	Protein	Molecular process	Biological process	FC	Regulation
1	*narZ*	Respiratory nitrate reductase 2 alpha chain	4 iron, 4 sulfur cluster binding, nitrate reductase activity	anaerobic electron transport chain, nitrate assimilation	94.99	down
2	*narW*	Probable nitrate reductase molybdenum cofactor assembly chaperone NarW	metallochaperone activity	chaperone-mediated protein complex assembly, nitrate assimilation, nitrate assimilation	70.67	down
3	*narV*	Respiratory nitrate reductase 2 gamma chain	electron carrier activity, metal ion binding	anaerobic electron transport chain, nitrate assimilation	37.67	down
4	*gcd*	Quinoprotein glucose dehydrogenase	magnesium ion binding, quinoprotein glucose dehydrogenase activity	Oxidoreductase	34.65	down
5	*narY*	Respiratory nitrate reductase 2 beta chain	3 iron, 4 sulfur cluster binding, electron carrier activity	anaerobic electron transport chain, nitrate assimilation	26.06	down
6	*ymdF*	Uncharacterized protein YmdF	Uncharacterized	Uncharacterized	26.04	down
7	*allB*	Allantoinase	allantoinase activity	allantoin assimilation pathway	24.86	up
8	*fbaB*	Fructose-bisphosphate aldolase class 1	fructose-bisphosphate aldolase activity	glycolytic process	21.92	down
9	*ybdR*	Uncharacterized zinc-type alcohol dehydrogenase-like protein YbdR	oxidoreductase activity	Oxidoreductase	20.01	down
10	*narU*	Nitrate/nitrite transporter NarU	nitrate transmembrane transporter activity	nitrate assimilation	19.26	down
11	*ompC*	Outer membrane protein C	Metal ion binding, Porin activity	Ion trasnport, Cellule response to DNA damage	18.34	down
12	*yciE*	Protein YciE	Uncharacterized	cellular response to DNA damage stimulus	17.43	down
13	*melA*	Alpha-galactosidase protein MelA	alpha-galactosidase activity, manganese ion binding, oxidoreductase	melibiose catabolic process	13.14	down
14	*csgD*	CsgBAC operon transcriptional regulatory protein	bacterial-type RNA polymerase core promoter proximal region sequence-specific DNA binding	positive regulation of DNA-templated transcription, initiation	9.27	down
15	*ydcW*	Gamma-aminobutyraldehyde dehydrogenase	1-pyrroline dehydrogenase activity	putrescine catabolic process	9.16	down
16	*ggt*	Gamma-glutamyltranspeptidase	gamma-glutamyltransferase activity	glutathione biosynthetic process	9.11	down
17	*yciG*	Uncharacterized protein YciG	Uncharacterized	bacterial-type flagellum-dependent swarming motility	8.47	down
18	*cadB*	cadaverine/lysine antiporter	lysine:cadaverine antiporter activity	amino acid transmembrane transport	8.06	down
19	*yncJ*	Uncharacterized protein YncJ	Uncharacterized	Uncharacterized	7.89	down
20	*ynfM*	Inner membrane transport protein YnfM	transporter activity	transmembrane transport	7.50	down
21	*ygbE*	Inner membrane protein YgbE	Uncharacterized	Uncharacterized	6.46	down
22	*fepB*	Ferrienterobactin-binding periplasmic protein	Uncharacterized	cellular response to DNA damage stimulus	5.53	up

23	*yghW*	Protein YghW	Uncharacterized	Uncharacterized	5.21	down
24	*csgE*	Curli production assembly protein CsgE	identical protein binding	biological adhesion, single-species biofilm formation	5.13	down
25	*ybfM*	Uncharacterized membrane protein YbfM	Uncharacterized	Uncharacterized	5.13	up
26	*gltA*	Citrate synthase Protein GltA	citrate (Si)-synthase activity	tricarboxylic acid cycle	4.66	up
27	*sdhC*	Succinate dehydrogenase cytochrome b556 subunit	electron carrier activity, metal ion binding	cytochrome complex assembly	4.57	up
29	*stpA*	DNA-binding protein StpA	DNA binding	regulation of transcription, DNA-templated	4.48	up
30	*sdhD*	Succinate dehydrogenase hydrophobic membrane anchor subunit	electron carrier activity	aerobic respiration	4.34	up
31	*glnP*	Glutamine transport system permease protein GlnP	amino acid transmembrane transporter activity	Amino-acid transport	4.30	up
32	*pphA*	Glycerol-3-phosphate acyltransferase	2,4,4-trimethyl-3-oxopentanoyl-CoA 2-C-propanoyl transferase activity	phospholipid biosynthetic process	4.24	down
33	*gltI*	Glutamate/aspartate import solute-binding protein	Amino-acid transport	amino acid transport	4.13	up
34	*eutC*	Ethanolamine ammonia-lyase light chain	cobalamin binding	cellular amino acid metabolic process	4.09	down
35	*yncC*	Putative metabolite transport protein YncC	Uncharacterized	metabolite transport	4.08	down
36	*putA*	Bifunctional protein PutA	1-pyrroline-5-carboxylate dehydrogenase activity	proline biosynthetic process	4.06	up
37	*gltJ*	Glutamate/aspartate transport system permease GltJ	L-glutamate ABC transporter	transporter activity	3.88	up
38	*fabA*	3-hydroxydecanoyl-[acyl-carrier-protein] dehydratase	3-hydroxydecanoyl-[acyl-carrier-protein] dehydratase activity	fatty acid biosynthetic process, lipid biosynthetic process	3.76	up
39	*entF*	Enterobactin synthase component F	2,3-dihydroxybenzoate-serine ligase activity, ATP binding	amino acid activation for nonribosomal peptide biosynthetic proces, enterobactin biosynthetic process	3.75	up
40	*htpX*	Protease HtpX	metalloendopeptidase activity	proteolysis	3.69	up
41	*ompF*	Outer membrane protein F	Drug transmembrane transporter activity, Porin activity	Ion transport, drug transmembrane transport	3.68	down
42	*glgP*	Glycogen phosphorylase	glycogen phosphorylase activity	glycogen catabolic process	3.37	down
43	*gltK*	Glutamate/aspartate import permease protein GltK	amino acid transmembrane transporter activity	Amino-acid transport	3.29	up
44	*lpxC*	UDP-3-O-acyl-N-acetylglucosamine deacetylase	lipid A biosynthetic process	deacetylase activity,	3.18	up
45	*modA*	Molybdate-binding periplasmic protein	ATPase-coupled molybdate transmembrane transporter activity	Transport	3.14	down

46	*csiE*	Stationary phase-inducible protein CsiE	Uncharacterized	regulation of transcription, DNA-templated	3.10	down
47	*narH*	Respiratory nitrate reductase 1 beta chain	3 iron, 4 sulfur cluster binding	nitrate assimilation	3.03	down
48	*tppB*	Dipeptide and tripeptide permease A	antibiotic transporter activity, dipeptide transmembrane transporter activity, symporter activity	antibiotic transport, hydrogen ion transmembrane transport	3.02	down
49	*spy*	Periplasmic chaperone Spy	ATP-independent chaperone mediated protein folding	Chaperone	2.96	down
50	*fabB*	3-oxoacyl-[acyl-carrier-protein] synthase 1	3-oxoacyl-[acyl-carrier-protein] synthase activity	fatty acid biosynthetic process	2.79	up
51	*araC*	Arabinose operon regulatory protein	transcription factor activity, sequence-specific DNA binding	regulation of transcription, DNA-templated	2.75	up
52	*mntH*	Divalent metal cation transporter MntH	manganese and cadmium ion transmembrane transporter activity	manganese and cadmium ion transport	2.67	up
53	*purB*	Adenylosuccinate lyase	(S)-2-(5-amino-1-(5-phospho-D-ribosyl)imidazole-4-carboxamido)succinate AMP-lyase (fumarate-forming) activity	'de novo' AMP biosynthetic process	2.65	down
54	*ybeX*	YbeX protein	flavin adenine dinucleotide binding, oxidoreductase activity, acting on CH-OH group of donors	Binding protein	2.60	down
55	*tqsA*	AI-2 transport protein TqsA	efflux transmembrane transporter activity	quorum sensing, single-species biofilm formation	2.57	up
56	*accC*	Biotin carboxylase	acetyl-CoA carboxylase activity	fatty acid biosynthetic process	2.54	down
57	*acrR*	HTH-type transcriptional regulator AcrR	transcription factor activity, sequence-specific DNA binding	negative regulation of transcription, DNA-templated	2.48	up
58	*fliD*	Flagellar hook-associated protein 2	Uncharacterized	bacterial-type flagellum-dependent cell motility	2.40	up
59	*hemH*	Ferrochelatase	ferrochelatase activity	heme biosynthetic process	2.30	down
60	*flgK*	Flagellar hook-associated protein 1	structural molecule activity	bacterial-type flagellum assembly	2.25	up
61	*otsA*	Alpha,alpha-trehalose-phosphate synthase [UDP-forming]	alpha,alpha-trehalose-phosphate synthase (UDP-forming) activity	response to osmotic stress, trehalose metabolism	2.24	down
62	*yehE*	Uncharacterized protein YehE	Uncharacterized	Uncharacterized	2.18	down
63	*ygiW*	Protein YgiW	Uncharacterized	single-species biofilm formation, cellular response to cadmium ion	2.14	up
64	*nrdD*	Anaerobic ribonucleoside-triphosphate reductase	ATP binding, ribonucleoside-triphosphate reductase activity	nucleobase-containing small molecule interconversion	2.03	down
65	*aroG*	Phospho-2-dehydro-3-deoxyheptonate aldolase, Phe-sensitive	3-deoxy-7-phosphoheptulonate synthase activity	cellular amino acid biosynthetic process	2.02	up
66	*yqjK*	Uncharacterized protein YqjK	Uncharacterized	Uncharacterized	2.02	down

Table S3B. Cluster analysis of overlapping OmpR targets upon osmotic stress

Cluster 1

Category	Term	Count	P_value	Benjamini	Genes
GOTERM_MF_DIRECT	GO:0008940~nitrate reductase activity	5	6.27E-06	3.88E-04	*narV, narH, narW, narY, narZ, narU, gltK, gltI,gltJ, ompF, ompC, gcd, ydcW, putA, nrdD,sdhC, accC, sdhD, fbaB, allB, gltA*
GOTERM_CC_DIRECT	GO:0009325~nitrate reductase complex	4	5.08E-05	7.12E-04	
KEGG_PATHWAY	eum00910:Nitrogen metabolism	6	1.78E-04	7.63E-03	
GOTERM_BP_DIRECT	GO:0042126~nitrate metabolic process	3	1.35E-03	4.36E-02	
KEGG_PATHWAY	eum02020:Two-component system	10	3.45E-03	7.15E-02	
UP_KEYWORDS	Oxidoreductase	9	2.64E-02	8.29E-01	
KEGG_PATHWAY	eum01120:Microbial metabolism in diverse environments	11	2.97E-02	3.51E-01	

Cluster 2

Category	Term	Count	P_value	Benjamini	Genes
GOTERM_BP_DIRECT	GO:0006099~tricarboxylic acid cycle	3	3.66E-02	4.60E-01	*gltA,accC, fbaB, sdhC, sdhD*
KEGG_PATHWAY	eum00020:Citrate cycle (TCA cycle)	3	1.16E-01	4.85E-01	
KEGG_PATHWAY	eum01200:Carbon metabolism	5	2.45E-01	7.01E-01	

Cluster 3

Category	Term	Count	P_value	Benjamini	Genes
KEGG_PATHWAY	eum01130:Biosynthesis of antibiotics	9	5.30E-02	3.74E-01	*gcd, sdhC, aroG, putA, accC, sdhD, fbaB, gltA, hemH, glgP, purB, fbaB, lpxC, eutC, fabB, fabA, otsA, ydcW, ggt, putA, aroG, nrdD, allB*
KEGG_PATHWAY	eum01110:Biosynthesis of secondary metabolites	11	9.83E-02	4.70E-01	
KEGG_PATHWAY	eum01100:Metabolic pathways	20	1.63E-01	5.72E-01	
KEGG_PATHWAY	eum01200:Carbon metabolism	5	2.45E-01	7.01E-01	

Cluster 4

Category	Term	Count	P_value	Benjamini	Genes
INTERPRO	IPR010065:Amino acid ABC transporter, permease protein, 3-TM domain, His/Glu/Gln/Arg/opine family	3	8.12E-03	7.56E-01	*gltK, gltJ, glnP, tppB, sdhD, mntH, ompF, ompC, ynfM, modA, gltI, fepB*
UP_KEYWORDS	Transport	8	1.41E-01	9.18E-01	
INTERPRO	IPR000515:Binding-protein-dependent transport systems inner membrane component	3	1.45E-01	1.00E+00	
UP_SEQ_FEATURE	domain:ABC transmembrane type-1	3	2.11E-01	1.00E+00	
UP_KEYWORDS	Cell membrane	7	2.57E-01	8.87E-01	

GOTERM_MF_DIRECT	GO:0005215~transporter activity	4	2.80E-01	9.99E-01	
KEGG_PATHWAY	eum02010:ABC transporters	6	3.84E-01	8.50E-01	
**Cluster 5**					
Category	Term	Count	P_value	Benjamini	Genes
UP_KEYWORDS	Transmembrane	18	1.50E-01	8.83E-01	*cadB, ynfM, tppB, narU, narV, ompC, ompF, gltK, htpX, gcd, yqjK, ygbE, sdhC, mntH, gltJ, tqsA, glnP, ynfM*
UP_SEQ_FEATURE	topological domain:Periplasmic	13	1.94E-01	1.00E+00	
UP_KEYWORDS	Membrane	18	2.57E-01	9.14E-01	
UP_KEYWORDS	Transmembrane helix	16	2.92E-01	8.74E-01	
UP_SEQ_FEATURE	topological domain:Cytoplasmic	12	2.97E-01	1.00E+00	
UP_SEQ_FEATURE	transmembrane region	16	3.71E-01	1.00E+00	
GOTERM_CC_DIRECT	GO:0016021~integral component of membrane	14	7.10E-01	1.00E+00	
**Cluster 6**					
Category	Term	Count	P_value	Benjamini	Genes
UP_KEYWORDS	Cell membrane	7	2.57E-01	8.87E-01	*gltK, htpX, tppB, sdhD, gltJ, mntH, glnP*
Category	Term	Count	P_value	Benjamini	
UP_KEYWORDS	Cell inner membrane	4	5.57E-01	9.50E-01	
GOTERM_CC_DIRECT	GO:0005886~plasma membrane	4	8.29E-01	1.00E+00	
**Cluster 7**					
Category	Term	Count	P_value	Benjamini	Genes
GOTERM_BP_DIRECT	GO:0006355~regulation of transcription, DNA-templated	4	3.22E-01	9.86E-01	*stpA, csgD, csiE, acrR, yncC, putA, araC*
UP_KEYWORDS	Transcription regulation	5	4.35E-01	9.45E-01	
UP_KEYWORDS	Transcription	5	4.53E-01	9.30E-01	
UP_KEYWORDS	DNA-binding	6	4.81E-01	9.33E-01	
GOTERM_BP_DIRECT	GO:0006351~transcription, DNA-templated	5	5.71E-01	9.99E-01	
GOTERM_MF_DIRECT	GO:0003700~transcription factor activity, sequence-specific DNA binding	3	7.83E-01	1.00E+00	
UP_SEQ_FEATURE	DNA-binding region:H-T-H motif	3	8.64E-01	1.00E+00	
GOTERM_MF_DIRECT	GO:0003677~DNA binding	5	9.10E-01	1.00E+00	
**Cluster 8**					
Category	Term	Count	P_value	Benjamini	Genes
UP_KEYWORDS	Zinc	3	4.39E-01	9.34E-01	*htpX, lpxC, allB, sdhD, hemH, narZ, melA, pphA*
UP_KEYWORDS	Metal-binding	6	5.36E-01	9.49E-01	
UP_KEYWORDS	Hydrolase	5	8.23E-01	9.97E-01	

Non-cluster					
Category	Term	Count	P_value	Benjamini	Genes
INTERPRO	IPR023614:Porin domain	3	8.12E-03	7.56E-01	*narZ, narU,, gltI, ompF, ompC, ybfM, ydcW, putA, nrdD,, accC, sdhD, fbaB, allB, gltA, ygiW, spy, modA, ggt, csgE, gltI, fepB, yehE, yncJ, fabB, fabA, aroG, purB,, spy, ggt, tqsA, hemH, glgP, mntH, lpxC, melA, ynfM, pphA, entF,, tppB*
GOTERM_MF_DIRECT	GO:0015288~porin activity	3	9.96E-03	2.67E-01	
UP_KEYWORDS	Signal	12	3.63E-02	7.05E-01	
KEGG_PATHWAY	eum00061:Fatty acid biosynthesis	3	4.60E-02	3.97E-01	
UP_SEQ_FEATURE	signal peptide	13	6.74E-02	1.00E+00	
KEGG_PATHWAY	eum01212:Fatty acid metabolism	3	8.82E-02	4.84E-01	
UP_KEYWORDS	Ion transport	3	1.25E-01	9.47E-01	
UP_KEYWORDS	Acyltransferase	3	2.20E-01	9.35E-01	
UP_KEYWORDS	Lyase	5	2.21E-01	9.05E-01	
UP_KEYWORDS	NAD	3	2.82E-01	8.88E-01	
GOTERM_MF_DIRECT	GO:0046872~metal ion binding	5	3.43E-01	9.98E-01	
INTERPRO	IPR020846:Major facilitator superfamily domain	3	3.67E-01	1.00E+00	
UP_KEYWORDS	Iron	3	3.94E-01	9.37E-01	
UP_SEQ_FEATURE	active site:Proton donor	3	3.95E-01	1.00E+00	
UP_SEQ_FEATURE	mutagenesis site	7	5.41E-01	1.00E+00	
KEGG_PATHWAY	eum00230:Purine metabolism	3	5.63E-01	9.49E-01	
UP_SEQ_FEATURE	sequence variant	3	0.11485	6.17E-01	
KEGG_PATHWAY	eum01230:Biosynthesis of amino acids	3	0.11485	7.52E-01	
UP_KEYWORDS	Transferase	7	0.26799	7.67E-01	
GOTERM_CC_DIRECT	GO:0005737~cytoplasm	3	0.11485	9.95E-01	

Table S4A. Description of overlapping OmpR targets responsive to both acid and osmotic stress

No	Gene	Protein	Molecular process	Biological process	FC	Regulation
1	*ymdF*	Uncharacterized protein YmdF	Uncharacterized	Uncharacterized	26.04	down
2	*ompC*	Outer membrane protein C	Metal ion binding, Porin activity	Ion trasnport, Cellule response to DNA damage	18.34	down
3	*yciF*	YciF	Uncharacterized	Cellular response to DNA damage stimulus	15.07	down
4	*yciG*	Uncharacterized protein YciG	Uncharacterized	bacterial-type flagellum-dependent swarming motility	8.47	down
5	*yncJ*	Uncharacterized protein YncJ	Uncharacterized	Uncharacterized	7.89	down
6	*ygbE*	Inner membrane protein YgbE	Uncharacterized	Uncharacterized	6.46	down
7	*gltA*	Citrate synthase Protein GltA	citrate (Si)-synthase activity	tricarboxylic acid cycle	4.66	up
8	*ompF*	Outer membrane protein F	Drug transmembrane transporter activity, Porin activity	Ion transport, drug transmembrane transport	3.68	down
9	*tppB*	Dipeptide and tripeptide permease A	antibiotic transporter activity, dipeptide transmembrane transporter activity, symporter activity	antibiotic transport, hydrogen ion transmembrane transport	3.02	down

Table S4B. Cluster analysis of overlapping OmpR targets upon both acid and osmotic

**Cluster 1**					
Category	Term	Count	P_value	Benjamini	Genes
UP_KEYWORDS	Transport	3	9.80E-02	7.87E-01	*tppB, ompF, ompC, ygbE*
UP_KEYWORDS	Transmembrane	4	2.16E-01	7.03E-01	
UP_KEYWORDS	Membrane	4	2.60E-01	6.76E-01	
UP_SEQ_FEATURE	topological domain:Periplasmic	3	3.27E-01	9.38E-01	
UP_SEQ_FEATURE	transmembrane region	3	5.39E-01	9.73E-01	

Table S5. Primers used in this study

Name	Sequences (5' to 3')
S. **Typhimurium 14028s**	
gltA::tetRA #F	TCCGGCAGTCTTAAGCAATAAGGCGCTAAGGAGACCGTAA TTAAGACCCACT<br>TTCACATT
gltA::tetRA #R	GTACCGGATGGCGAGGGTTGCGTCGCCATCCGGTTGTCAA<br>CTAAGCACTTGTCTCCTG
gltA EcoR1#1F	GGCCGAATTC ATGGCTGATACAAAGGCAAAA
gltA HindIII#1R	GGCCAAGCTT CGCTTCAGCGCCGATTTAAAA
gltA F	GTAGCCAGAAAGACGACGGG
gltA R	TTTGTATCAGCCATTTACGGT
E. coli **1655**	
gltAEC::tetRA #F	TCCGGCAGTCTTACGCAATAAGGCGCTAAGGAGACCTTAA<br>TTAAGACCCACTTTCACATT
gltAEC::tetRA #R	GAACGGCGGGTTAAAATATTTACAACTTAGCAATCAACCA<br>CTAAGCACTTGTCTCCTG
gltA EcoR1#1F	GGCCGAATTC ATGGCTGATACAAAAGCAAAA
gltA HindIII#1R	GGCCAAGCTT ACGCTTGATATCGCTTTTAAA
gltA F	TGCTGTTCTTATTATTCCCT
gltA R	ATCAGCCATTTAAGGTCTCC

FIGURE 1

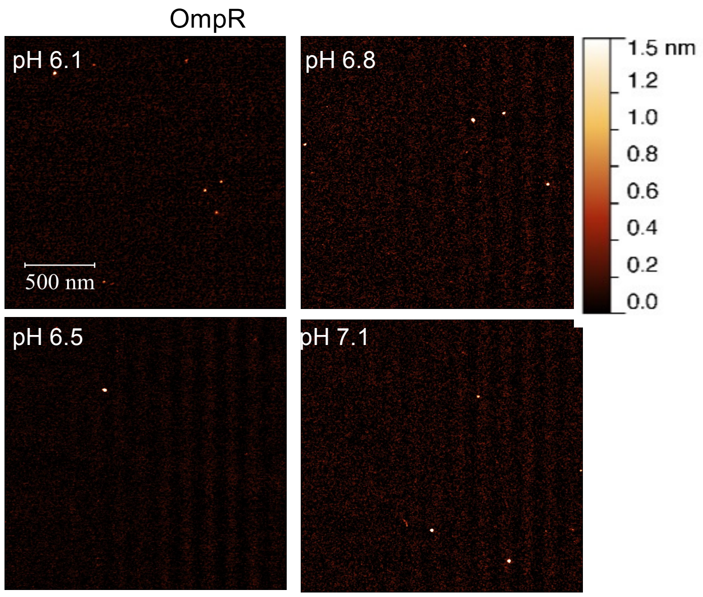

Figure S1. AFM images of OmpR in the absence of DNA. No aggregation is evident at a range of pH values from 6.1-7.1. 30 nM OmpR was deposited on the mica at various pH values as indicated.
